# *Kalicephalus* (*Occipitodontus*) *colossus* (Nematoda: Diaphanocephalidae): a new hookworm from *Naja kaouthia* in central Thailand

**DOI:** 10.1017/S0031182026102248

**Published:** 2026-06

**Authors:** Vachirapong Charoennitiwat, Sila Viriyautsahakul, Urusa Thaenkham, Kittipong Chaisiri, Napat Ratnarathorn

**Affiliations:** 1 Laboratory of Helminth Biodiversity and Drug Development, Department of Helminthology, Faculty of Tropical Medicine, Mahidol University, Bangkok, Thailand; 2 Applied Animal Science Laboratory, Department of Biology, Faculty of Science, Mahidol University, Bangkok, Thailand

**Keywords:** integrative taxonomy, monocled cobra (*Naja kaouthia*), new nematode species, phylogenetics, snake parasite

## Abstract

The genus *Kalicephalus* Molin, 1861 comprises hookworms parasitizing the gastrointestinal tract of reptiles, particularly snakes, and currently includes 34 recognized species worldwide. Despite their diversity, the taxonomy of the genus remains poorly resolved because most species have been described primarily from morphology and molecular data remain limited. During a helminthological survey of monocled cobras (*Naja kaouthia*) from central Thailand, large hookworms were recovered from the oesophagus that corresponded morphologically to the group traditionally assigned to *Occipitodontus* but differed from previously described species. Using an integrative taxonomic approach combining light microscopy, scanning electron microscopy, morphometric analyses and molecular phylogenetic analyses based on nuclear 18S ribosomal RNA (18S rRNA) and mitochondrial cytochrome c oxidase subunit I (*COI*) sequences, *Kalicephalus colossus* sp. nov. is described herein. The new species is distinguished by its exceptionally large body size, posteriorly projecting buccal capsule, equal alate spicules, lobate copulatory bursa and oesophageal localization. Multivariate analyses revealed clear sexual dimorphism without evidence of discrete morphotypes. Phylogenetic analyses recovered *K. colossus* sp. nov. as a distinct lineage within *Kalicephalus*, with genetic divergences of 1.1–1.4% for 18S rRNA and 12–13% for *COI* relative to congeners. The parasite was detected in 18% of examined hosts, with infection intensities ranging from 1 to 48 individuals per cobra, and was recorded only from suburban localities surrounding Bangkok. No obvious gross pathological lesions were observed during necropsy. The discovery of *K. colossus* sp. nov. expands current knowledge of reptile hookworm diversity in Southeast Asia and highlights the importance of integrative taxonomy for resolving parasite biodiversity.

## Graphical abstract

**Figure:**
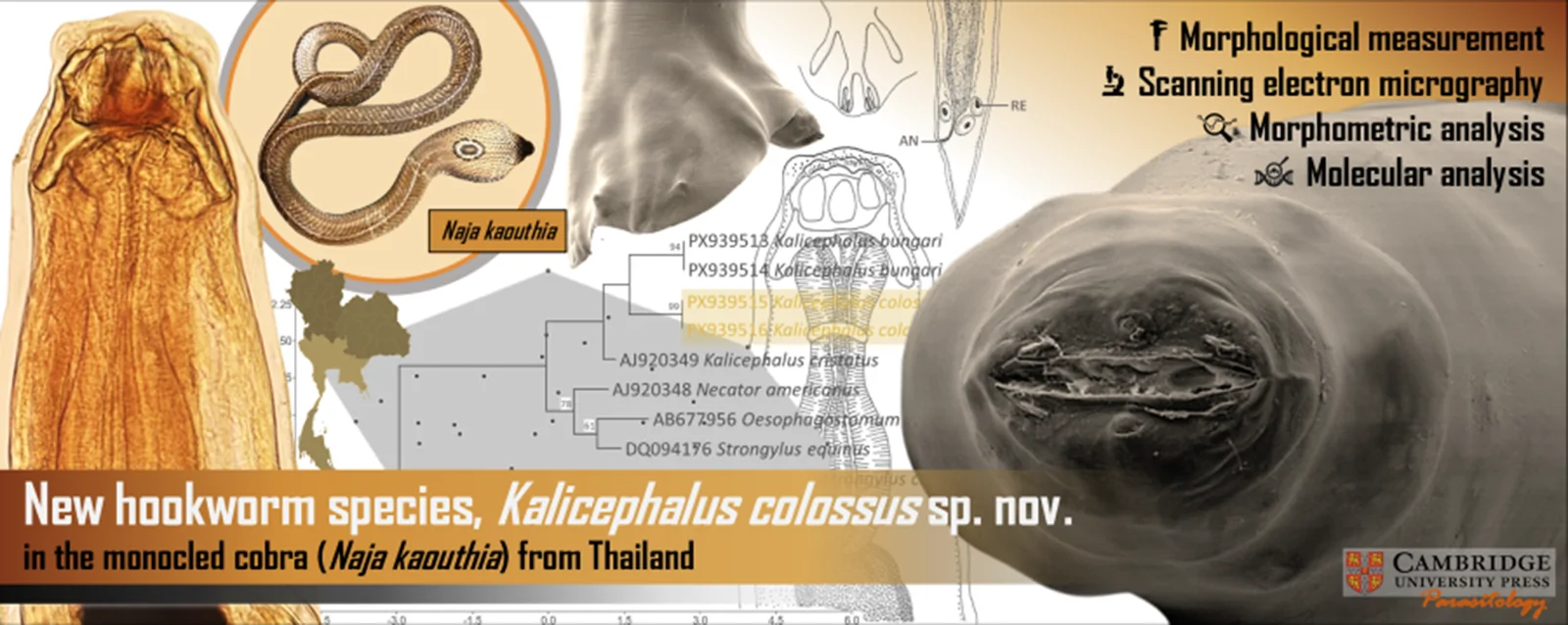

## Introduction

The genus *Kalicephalus* Molin, 1861 comprises a diverse group of hookworms parasitizing the gastrointestinal tract of snakes and lizards and is cosmopolitan in distribution. At present, 34 species are recognized globally (Chandel and Dolma, 2025; Haick et al., 2025). Despite this recognized diversity, the taxonomic history of *Kalicephalus* has been long and complex. Within the family Diaphanocephalidae, species now placed in *Kalicephalus* were originally described as the genus *Occipitodontus* by Ortlepp (1923), based on the presence of posteriorly extended chitinoid elements of the buccal capsule. Schad (1962) later reduced *Occipitodontus* to a subgenus of *Kalicephalus*. Subsequent taxonomic treatments synonymized *Occipitodontus* with *Kalicephalus*; however, some authors have continued to recognize it as a distinct subgenus (*sensu* Anderson et al., 2009; Kuzmin et al., 2013). Nevertheless, the morphological characters emphasized by Ortlepp remain taxonomically informative and useful for distinguishing species traditionally assigned to *Occipitodontus* within *Kalicephalus* (Anderson et al., 2009).

Southeast Asia and the broader Oriental biogeographic region represent a major centre of *Kalicephalus* diversity, harbouring 17 of the 34 recognized species (Schad, 1962; Zhang et al., 2011; Kuzmin et al., 2013; Chandel and Dolma, 2025; Haick et al., 2025). These include *Kalicephalus fimbriatus* (Ortlepp, 1923) (India and Malaya); *Kalicephalus assimilis* (Wu and Hu, 1938) (Hainan Island, China; Malaya; Borneo and Java, Indonesia); *Kalicephalus bungari* (MacCallum, 1918) (Thailand; Sumatra and Java, Indonesia; China; and Japan); *Kalicephalus costatus indicus* (Ortlepp, 1923) (India; Malaya; China; Australia; and Java, Indonesia); *Kalicephalus viperae* (Rudolphi, 1819) (China; Taiwan; Okinawa, Japan; and Malaya); *Kalicephalus willeyi* Linstow, 1904 (Komodo Island, Indonesia and Papua New Guinea); *Kalicephalus sinensis* (Hsü, 1934) (China and Taiwan); *Kalicephalus variabilis* (Schad, 1962) (China and Taiwan); *Kalicephalus laticaudae* (Yamaguti, 1935) (Ryukyu Islands, Japan; and Taiwan); *Kalicephalus brachycephalus* Maplestone, 1931 (China and Korea); *Kalicephalus guangdongensis* Zhang, 2011 (Guangdong, China); *Kalicephalus truncatus* (Schad, 1962) (Southeast Asia and the East Indies); *Kalicephalus alatospiculus* (Yeh, 1957) (the Oriental region); *Kalicephalus burseyi* Kuzmin, 2013 (Luzon Island, the Philippines); *Kalicephalus longispicularis* (Schad, 1962) (Southeast Asia; East Indies; and the Philippines); *Kalicephalus schadi* Ogden, 1966 (India); and *Kalicephalus bilaspurense* Chandel and Dolma, 2025 (India). Despite this high species diversity, systematic information on *Kalicephalus*, particularly molecular phylogenetic data, remains unevenly updated, thereby hindering accurate species delimitation and comparative analyses.

Based on variation in the morphology of the buccal capsule, uterus, spicules and copulatory bursa, *Kalicephalus* has been subdivided into 6 subgenera (Schad, 1962; Lichtenfels, 1980; Baker, 1987; Anderson et al., 2009). Anderson and colleagues (2009) retained a classification recognizing *Occipitodontus* Ortlepp, 1923; *Schadius* Lichtenfels, 1980; *Kalicephaloides* Yeh, 1956; *Rectiphiloides* Schad, 1962; *Variabiliformis* Schad, 1962; and *Inermiformis* Schad, 1962. Subsequent taxonomic revisions (e.g., Zhang et al., 2011), however, synonymized *Schadius* with the nominotypical subgenus *Kalicephalus* (*Kalicephalus*) Molin, 1861, whereas Haick et al. (2025) questioned the evolutionary validity of these subgeneric arrangements. Nevertheless, these subgenera remain useful as practical morphological frameworks for species identification and comparative taxonomy. Among them, *Occipitodontus* is particularly distinctive in possessing a rudimentary corona radiata, characteristic copulatory bursal morphology and a buccal capsule bearing prominent posteriorly directed dorsal and ventral plates. Species traditionally assigned to this subgenus primarily parasitize the gastrointestinal tract of Oriental and Australasian snakes and generally differ from other *Kalicephalus* taxa in their more elaborate cephalic and buccal structures (Anderson et al., 2009).

Owing to its distinctive morphology, the subgenus *Occipitodontus* currently includes only 3 species, *K. fimbriatus, K. assimilis* and *K. gigantaus*, which are characterized by posteriorly prolonged chitinoid elements of the buccal capsule that define the subgenus (*sensu* Schad, 1962; Anderson et al., 2009). During a helminthological survey of monocled cobras (*Naja kaouthia*) from central Thailand, several large hookworms were recovered from the oesophagus. These specimens exhibited the diagnostic characters of *Kalicephalus* (*Occipitodontus*), yet differed markedly from all previously described species by having a noticeably larger size and a distinct morphology of the posterior chitinoid plates of the buccal capsule, indicating the presence of an undescribed taxon (*sensu* Anderson et al., 2009).

The discovery of these specimens is particularly relevant given the ecology of the host species. The monocled cobra (*N. kaouthia*) is widespread and abundant throughout the Oriental region, including Thailand (Stuart and Wogan, 2012; Ratnarathorn et al., 2019, 2023). As a member of the family Elapidae, it is of significant medical importance and occupies a high trophic position, preying on amphibians, reptiles, small mammals and birds (Cox et al., 2012). This particular ecology as an active terrestrial predators increases exposure to trophically transmitted parasitic helminths and soil-transmitted helminths, including *Kalicephalus* (Charoennitiwat et al., 2023; Ratnarathorn et al., 2025). In addition, the tolerance of human-modified environments of *N. kaouthia* renders it ecologically important in anthropogenic landscapes (Ratnarathorn et al., 2024, 2026), where it may serve as a reservoir for parasites and other pathogens in urban to suburban areas (Ratnarathorn et al., 2025).

From a health and conservation perspective, infections by *Kalicephalus* nematodes can induce a range of pathological effects in snakes, from mild enteritis to severe clinical manifestations such as anorexia, dyspnoea and dysentery (Junker et al., 2009; Liu et al., 2019). These hookworms attach firmly to the gastrointestinal mucosa and may cause significant morbidity at high infection intensities, particularly in captive or intensively managed snakes (Chaiyabutr and Chanhome, 2002; Kavitha et al., 2014; Vasaruchapong et al., 2017). Therefore, accurate parasite identification is essential for effective health management, disease control and conservation, especially given the wildlife trade and increasing human–snake interactions (Chaiyabutr and Chanhome, 2002; Vasaruchapong et al., 2017; Charoennitiwat et al., 2024a, 2024b).

In light of these taxonomic, ecological and host–parasite considerations, the present study aims to formally describe a new species within its appropriate systematic context, thereby facilitating future taxonomic revisions and accurate species identification. An integrative approach, combining detailed morphological examination with molecular phylogenetic analyses of nuclear 18S ribosomal RNA (18S rRNA) and mitochondrial cytochrome c oxidase subunit I (*COI*) sequences, provides robust evidence supporting the recognition of *Kalicephalus colossus* sp. nov., Charoennitiwat – the 35th recognized species of the genus. This study contributes to a clearer understanding of *Kalicephalus* diversity, host associations and evolutionary relationships within the genus.

## Materials and methods

### Morphological study

Seventy-eight carcasses of wild monocled cobras that were accidentally killed or died naturally in central Thailand were provided by snake rescue teams or local villagers and transferred to the Applied Animal Science Laboratory, Department of Biology, Faculty of Science, Mahidol University. The specimens were dissected to examine the oesophagus for parasitic helminth infections, following the protocol described by Ratnarathorn and Kongrit (2025). After dissection, the organs were placed in Petri dishes containing 0.85% normal saline and examined under stereomicroscopes (Olympus SZ30 and SZ51, Japan). Parasites were observed and isolated using fine micro-dissecting needles, with particular emphasis on hookworms. All recovered hookworms were counted and preserved in 70% ethanol at −20 °C for subsequent morphological analyses. Additionally, 4 specimens were fixed in 2.5% glutaraldehyde for scanning electron microscopy (SEM), and 4 adult specimens (2 males and 2 females) were preserved for molecular analyses.

**Figure 1.**
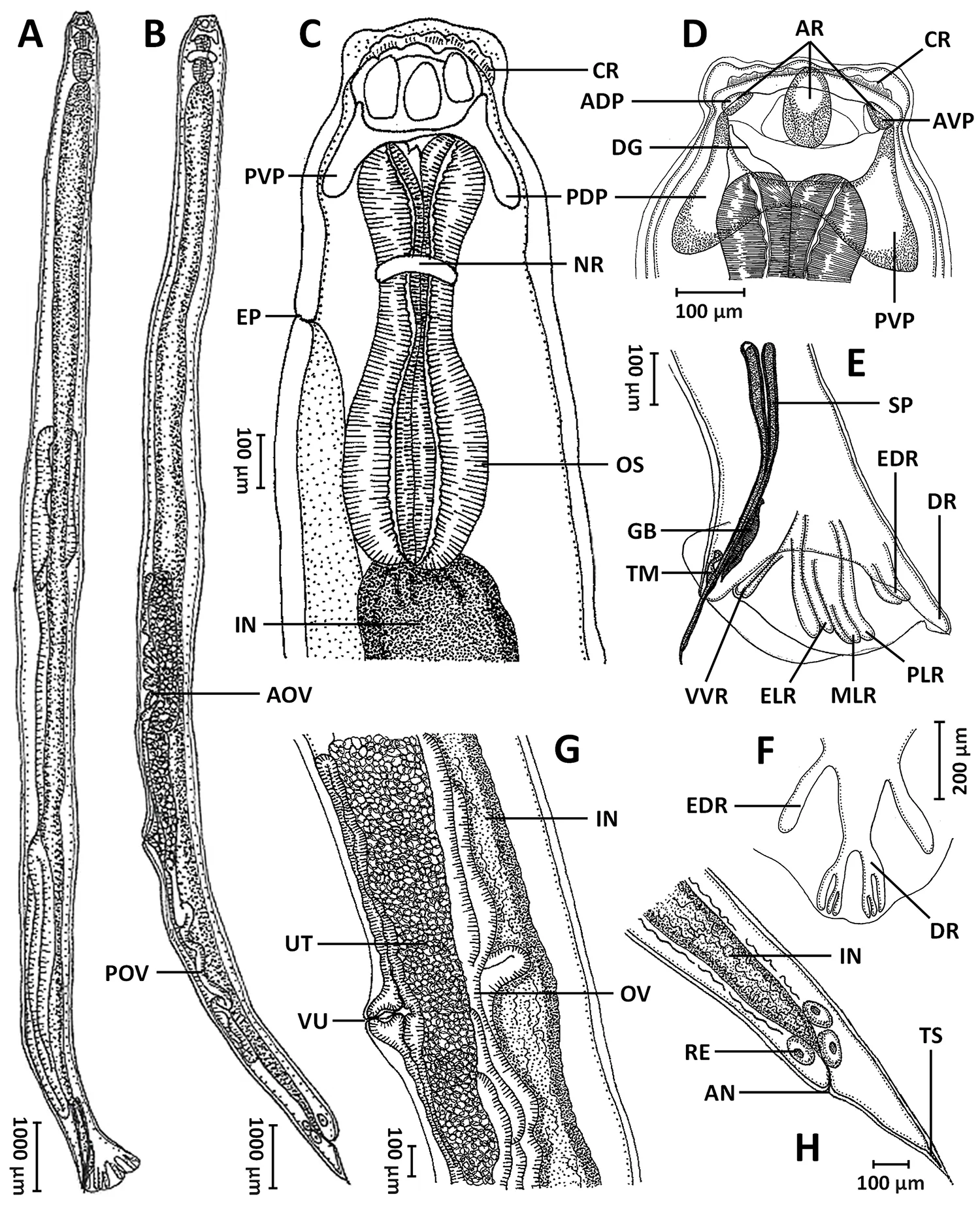
Line drawings of *Kalicephalus colossus* sp. nov.: (A) entire body of male; (B) entire body of female; (C) anterior end of the body, lateral view; (D) buccal capsule, lateral view; (E) posterior region of male, dorsal view; (F) copulatory bursa, ventral view; (G) vulvar region, lateral view; and (H) posterior end of female, lateral view. Abbreviations: ADP, anterior dorsal plate; AN, anus; AOV, anterior oviduct; AR, anterior chitinoid ridge; AVP, anterior ventral plate; CR, corona radiata; DG, dorsal gutter; DR, dorsal ray; EDR, externodorsal ray; ELR, externolateral ray; EP, excretory pore; GB, gubernaculum; IN, intestine; MLR, mediolateral ray; NR, nerve ring; OS, oesophagus; OV, oviduct; PDP, posterior dorsal plate; PLR, posterolateral ray; POV, posterior oviduct; PVP, posterior ventral plate; RE, rectal gland; SP, spicule; TM, telemon; TS, terminal spine; UT, uterus; VU, vulva and VVR, ventroventral ray.

From 5 infected cobra hosts (out of 78 examined individuals), a total of 15 male and 15 female intact hookworm specimens preserved in 70% ethanol were subjected to detailed morphological examination using an inverted microscope (Zeiss Primovert, Germany) equipped with a Zeiss Axiocam and ZEN 2 blue edition software. Morphological measurements followed the criteria and definitions outlined by Haick et al. (2025) and Chandel and Dolma (2025), and all measurements were recorded in micrometres (µm). Line drawings illustrating selected characters of both male and female specimens were prepared using a light microscope equipped with a camera lucida (Leitz, Wetzlar, Germany) (see Figure 1 for illustrations).

**Figure 2.**
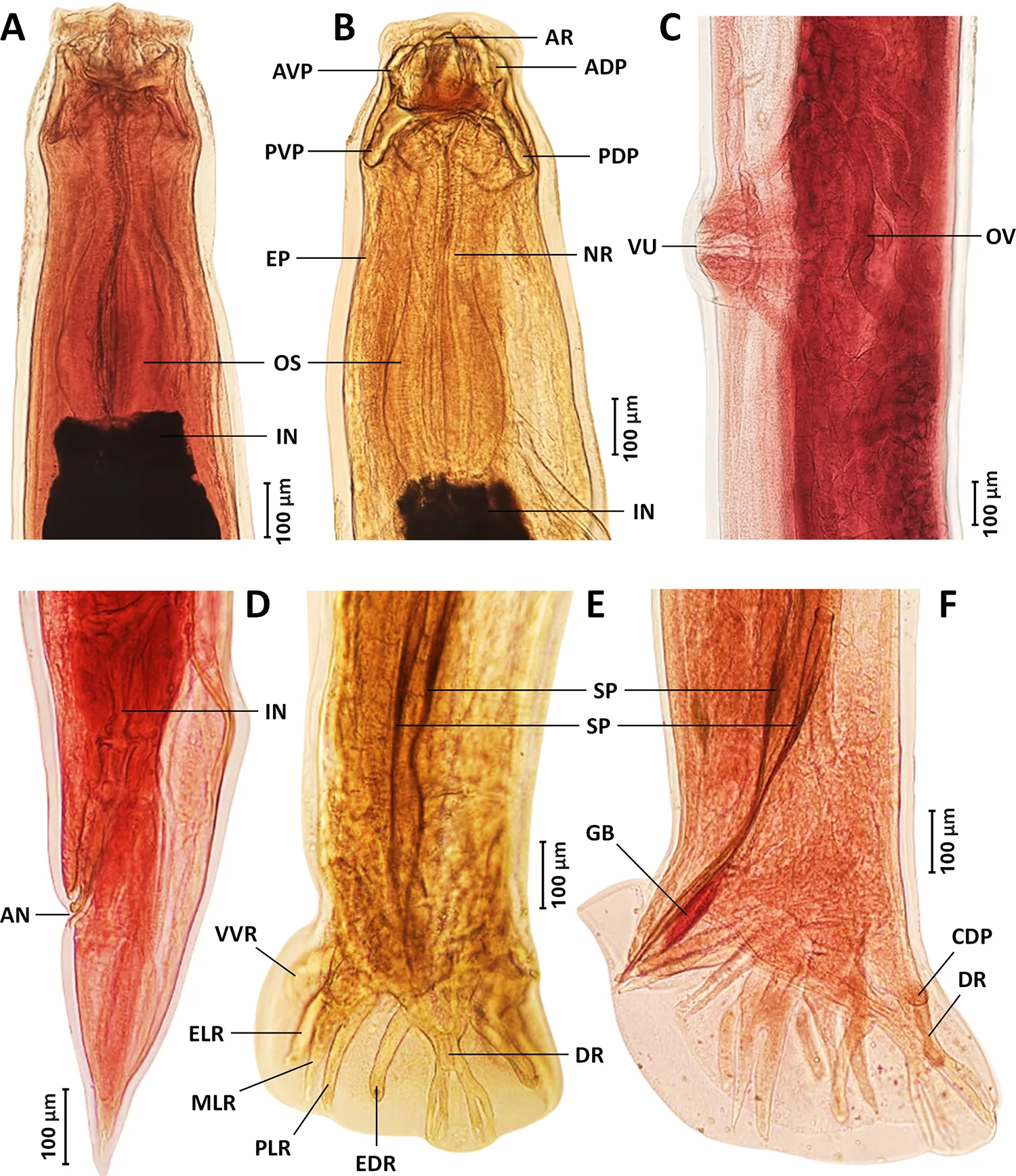
Permanent acetocarmine-stained (A, C, D and F) and glycerin-mounted, unstained (B and E) specimens of *Kalicephalus colossus* sp. nov.: (A) anterior region, dorsal view; (B) anterior region, lateral view; (C) vulvar region, lateral view; (D) posterior region of female, lateral view; (E) posterior region of male, dorsal view; and (F) posterior region of male, lateral view. Abbreviations: ADP, anterior dorsal plate; AN, anus; AR, anterior chitinoid ridge; AVP, anterior ventral plate; CDP, caudal papilla; DR, dorsal ray; EDR, externodorsal ray; ELR, externolateral ray; EP, excretory pore; GB, gubernaculum; IN, intestine; MLR, mediolateral ray; NR, nerve ring; OS, oesophagus; OV, oviduct; PDP, posterior dorsal plate; PLR, posterolateral ray; PVP, posterior ventral plate; SP, spicule; VU, vulva; and VVR, ventroventral ray.

Two well-preserved specimens of each sex were selected for the preparation of permanent slides designated as the holotype and paratypes. These specimens were stained with acetocarmine and dehydrated through a graded ethanol series (70, 80, 90, 95 and 100%), with each step lasting 45 min. Following dehydration, the specimens were transferred through a graded glycerin series (10, 25, 50, 70 and 100%) to facilitate gradual clearing and equilibration in glycerin. The specimens were then mounted in glycerin jelly on glass slides with coverslips and subsequently double-mounted with Permount™ to ensure long-term preservation and slide stability. Slides were cured at 60 °C for several days (Charoennitiwat et al., 2025a, 2025b). The remaining specimens (*n* = 26) were mounted temporarily in lactophenol for comparative observation (see Figure 2 for illustrations).

**Figure 3.**
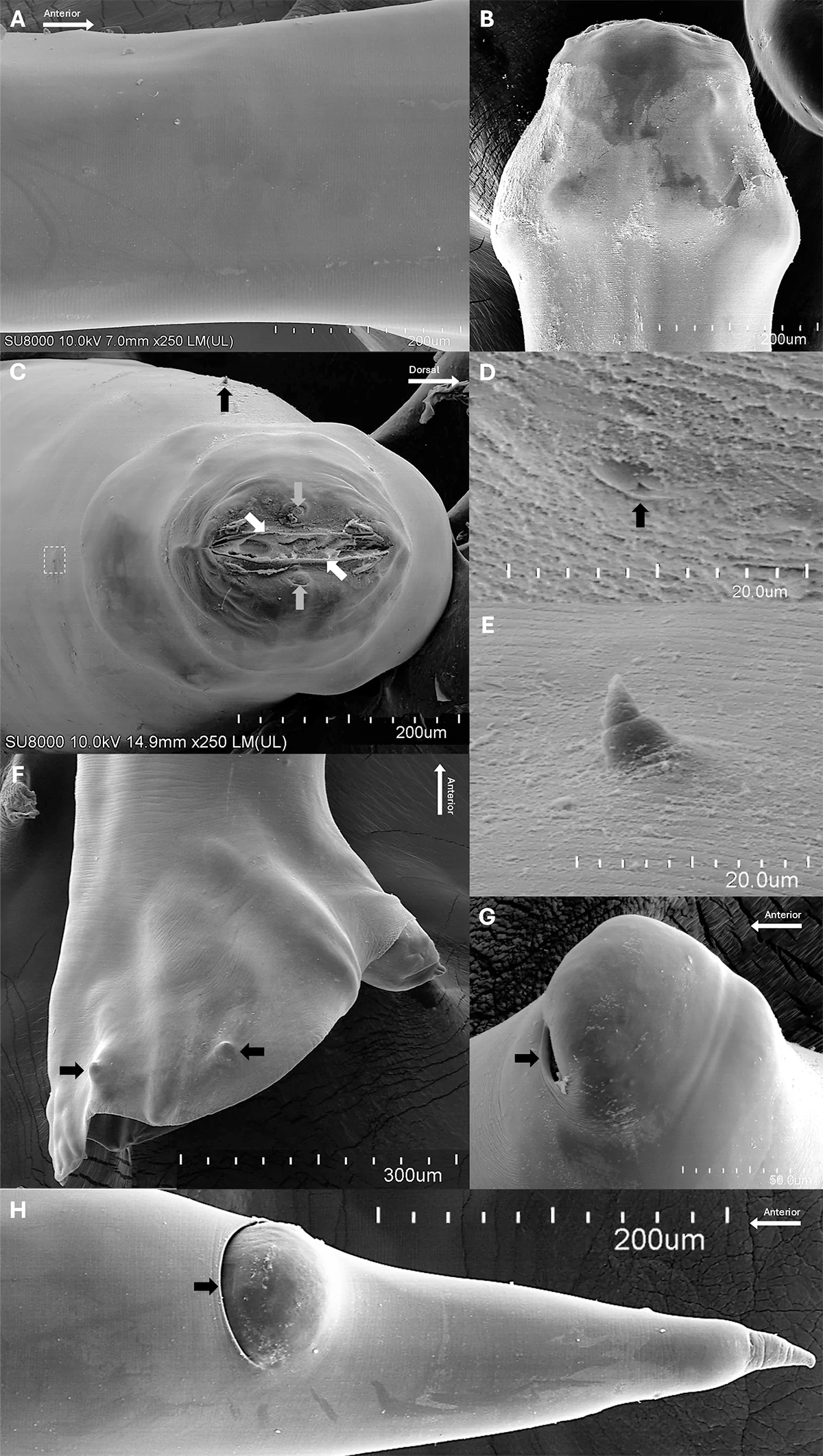
Scanning electron micrographs of *Kalicephalus colossus* sp. nov.: (A) cuticular surface at mid-body; (B) cephalic region, lateral view; (C) oral opening with lip surfaces bearing frilled corona radiata (white arrow) and showing the positions of the cervical papilla (black arrow), amphids (grey arrow) and excretory pore (dashed box), apical view; (D) excretory pore (black arrow), enlarged from panel C; (E) cervical papilla; (F) copulatory bursa of male with caudal papillae (black arrows), dorsal view; (G) vulva with the aperture directed anteriorly (black arrow); and (H) posterior region of female with anus (black arrow).

For SEM, specimens were fixed in 2.5% glutaraldehyde in 0.1 M sucrose phosphate buffer, followed by post-fixation in 1% osmium tetroxide in the same buffer. Samples were dehydrated through an ethanol series and dried using a critical point dryer (CPD300 Auto; Leica, Wetzlar, Germany). Dried specimens were sputter-coated with gold (Q150R PLUS; Quorum, East Sussex, UK). Specimen preparation was carried out at the Department of Tropical Pathology, Faculty of Tropical Medicine, Mahidol University, and SEM imaging was performed using a Hitachi SU8010 microscope at the Central Instrument Facility, Faculty of Science (Phaya Thai), Mahidol University (see Figure 3 for illustrations).

**Figure 4.**
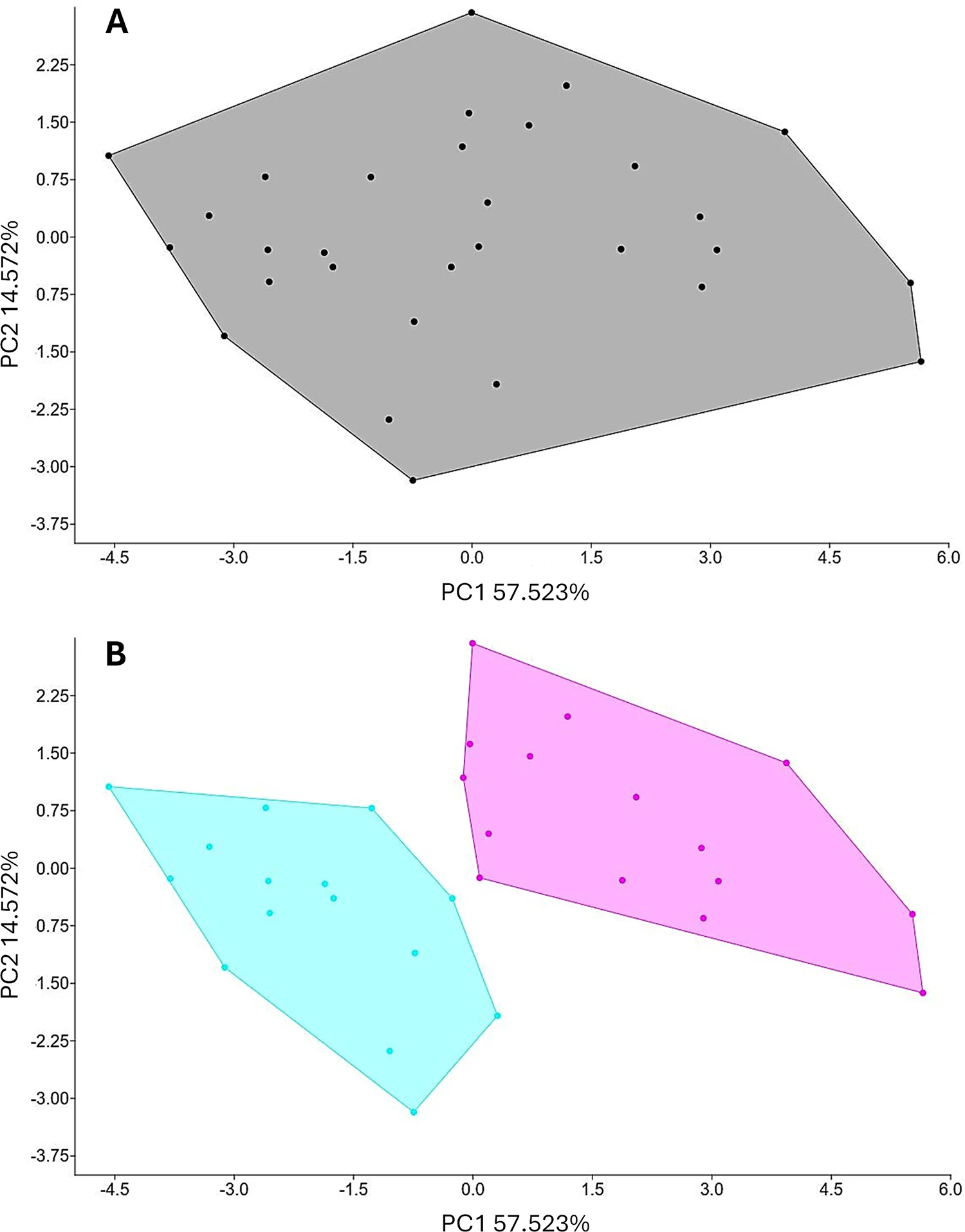
Principal component analysis (PCA) of *Kalicephalus colossus* sp. nov. specimens. The PCA was conducted using 12 morphological characters, explaining 72% of the total variance. (A) Black dots and lines represent individual specimens and their clustering; and (B) grouping by sex, with pink dots and lines indicating female specimens and their clustering and blue dots and lines indicating male specimens and their clustering. Figure 4 long description.

To evaluate morphological variation among the 30 helminth specimens, 12 quantitative characters were measured and expressed in micrometres (μm). Measurements are presented as minimum–maximum ranges. The characters measured included body length, maximum body width, distance from the anterior end to the end of the oesophagus, dorsoventral head diameter, buccal capsule depth, distance from the anterior end to the nerve ring, oesophagus length, oesophagus width, oesophagus width at the oesophago–intestinal junction, distance from the anterior end to the excretory pore, distance from the anterior end to the cervical papillae and body cuticle thickness (see Table 1 and Supplementary Table S1). A variance–covariance matrix was constructed to analyse these variables. To further assess sexual dimorphism, sex-specific morphological characters were included in separate analyses. For males, additional characters comprised spicule length and width, gubernaculum length and width and telamon length and width, resulting in a total of 18 measured characters. For females, vulvar position ratio (pre-vulvar: post-vulvar), tail length and egg length and width were included, yielding a total of 16 characters. The resulting multivariate datasets were analysed using principal component analysis (PCA) implemented in PAST version 4.06b (Hammer et al., 2001). Both variance–covariance and correlation matrices were used to generate 2D scatter plots illustrating morphological variation among specimens, with associated percentages of explained variance (e.g., Viriyautsahakul et al., 2025). In addition, discriminant analysis (DA) was applied to identify morphological traits contributing most strongly to differentiation among groups (see Figure 4 for illustrations).

**Table 1.** Comparison of characteristics among males and females across *Kalicephalus* (*Occipitodontus*) species Table 1 long description.

Species	*Kalicephalus colossus*	*Kalicephalus fimbriatus*	*Kalicephalus assimilis*	*Kalicephalus assimilis*	*Kalicephalus giganteus*
Reference	Present study	Schad (1962)	Schad (1962)	Purwaningsih (2011)	Schad (1962)
Host species	*Naja kaouthia*	*Bungarus fasciatus, Ophiophagus bungarus*	*Ophiophagus bungarus*	*Ophiophagus bungarus*	*Liasis papuanus*
Locality	Thailand	India & Malaya	Hainan Island, Malaya & Celebes	Indonesia	New Guinea
Organ	Oesophagus	Stomach	Intestine	Intestine	Intestine
**Male**	*n* = 15	*n* = 2	*n* = 10	N/A	*n* = 8
Body length	13 345–17 680	9520–12 000	7310–8790	6290–8180	13 880–16 350
Maximum width	582–703	400–440	260–300	420–450	450–620
Anterior end to oesophagus end	891–1075	–	–	–	–
Dorsoventral head diameter	376–456	440	260–290	160–180	330–380
Buccal capsule depth	204–271	230–260	130–150	110–150	230–260
Nerve ring from anterior end	387–493	370–410	290–320	270–320	370–410
Oesophagus length	634–850	590–670	470–550	520–580	600–720
Oesophagus width	250–312	220–310	190–220	–	210–260
Oesophagus width at OI junction	139–192	–	–	–	–
Excretory pore from anterior end	468–604	390–500	470–530	410–480	680–960
Cervical papillae from anterior end	526–705	440	520–590	–	710–1060
Body cuticle width	22–39	–	–	–	–
Spicule length	545–705	520	440–500	410–550	830–950
Spicule width	18–35	–	–	–	–
Gubernaculum length	101–146	–	–	–	–
Gubernaculum width	3–15	–	–	–	–
Telamon length	56–95	–	–	–	–
Telamon width	17–54	–	–	–	–
**Female**	*n* = 15	*n* = 5	*n* = 10	N/A	*n* = 6
Body length	14 854–21 159	11 400–14 630	8770–10 690	7350–9570	17 530–21 340
Maximum width	680–903	460–570	320–380	420–450	790–860
Anterior end to oesophagus end	990–1165	–	–	–	–
Dorsoventral head diameter	451–503	490–550	300–350	–	430–510
Buccal capsule depth	227–285	230–280	140–170	140–190	290–340
Nerve ring from anterior end	447–604	410–450	320–380	340–390	480–560
Oesophagus length	738–920	680–750	500–590	510–580	750–850
Oesophagus width	265–342	270–310	210–260	–	260–330
Oesophagus width at OI junction	132–221	–	–	–	–
Excretory pore from anterior end	537–720	410–480	510–620	310–480	790–980
Cervical papillae from anterior end	601–771	480–510	530–640	–	830–1070
Body cuticle width	23–45	–	–	–	–
Vulva ratio (pre:post)	1.67–2.12	1.7–2.0	1.9–2.4	1.8–2.1	1.4–1.7
Tail length	311–498	290–370	310–370	330–380	470–620
Egg length	57–88	–	–	68–75	–
Egg width	41–54	–	–	51–71	–

### Molecular and phylogenetic study

For molecular analysis, DNA was extracted from 2 specimens using the Geneaid genomic DNA mini kit (Geneaid Biotech Ltd, Taipei, Taiwan), following the manufacturer’s instructions. Amplification targeted partial sequences of 2 genetic markers: nuclear 18S rRNA and mitochondrial *COI* gene. These publicly reference-available markers were selected for their proven utility in nematode systematics and molecular identification. The primers used, expected amplicon lengths and other amplification details are provided in Supplementary Table S2 (Holterman et al., 2006; Chan et al., 2020; Thaenkham et al., 2022). PCR amplification was conducted in a final reaction volume of 30 μL.

Reactions were performed using a T100™ thermocycler (Bio-Rad, California, USA) in a 30 µL mixture containing 15 µL of 2X i-Taq Master Mix (iNtRON Biotechnology, Gyeonggi, South Korea), 10 µM of each primer and 1 ng µL^−1^ of template DNA. Thermal cycling conditions are also provided in Supplementary Table S2. PCR amplicons were visualized on a 1% agarose gel stained with SYBR Safe™ (Thermo Fisher Scientific, Massachusetts, USA). Selected PCR products were sequenced using Fast Next-Generation Sequencing (Tsingke, Beijing, China). The resulting nucleotide sequences were deposited in the NCBI database under the following accession numbers: PX939515 and PX939516 (18S rRNA) and PX940603 and PX940605 (*COI*).

**Figure 5.**
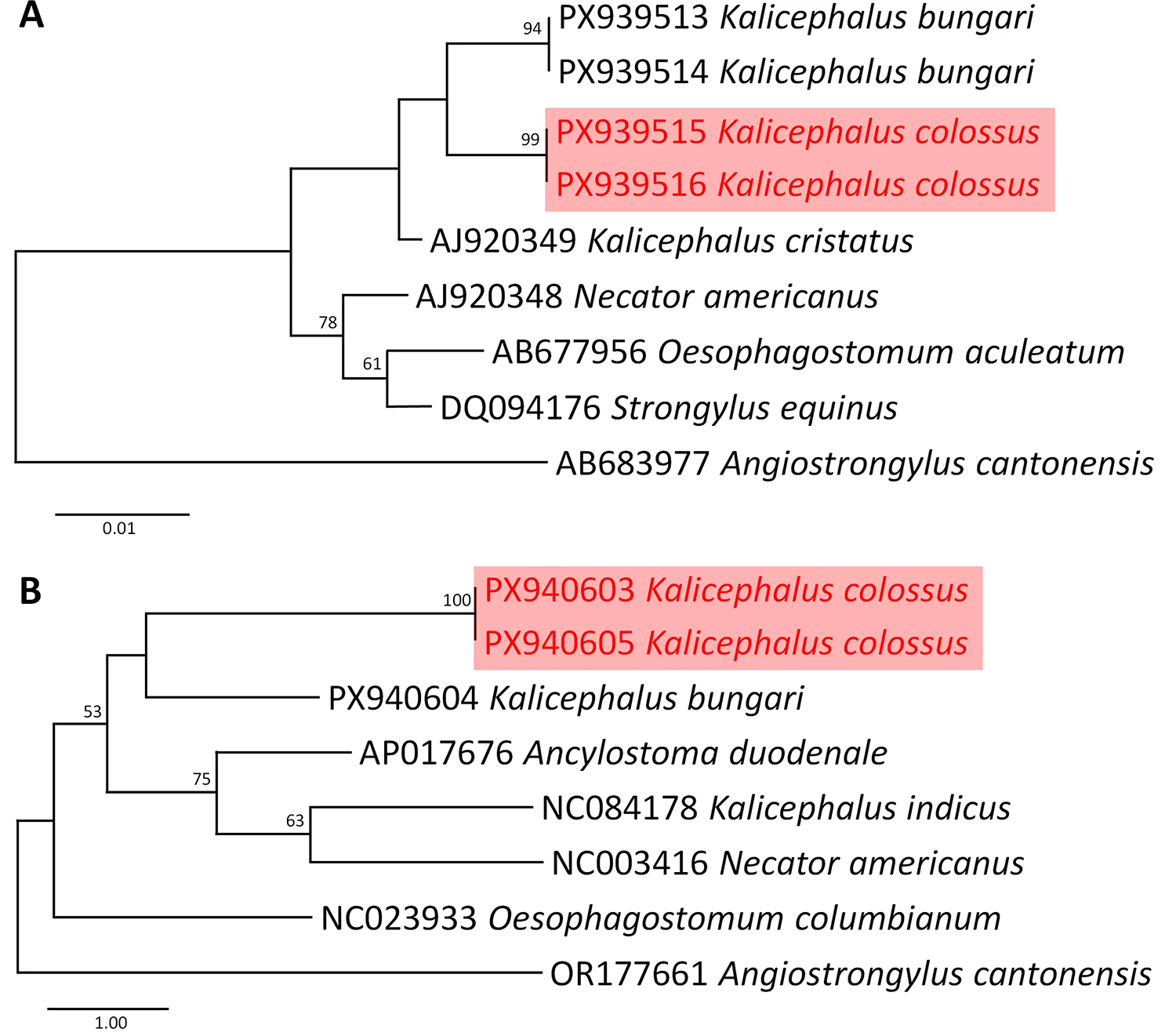
Phylogenetic analyses of available sequences of *Kalicephalus* and other strongylid species, with *Angiostrongylus cantonensis* as an outgroup, based on the 18S rRNA (A) and *COI* (B) genes. Analyses were performed using the maximum likelihood method in MEGA 12. Branch length scale bars indicate the number of substitutions per site, and node values represent bootstrap support from maximum likelihood and Bayesian inference. *Kalicephalus colossus* sp. nov. identified in this study is highlighted in red (font/box). Figure 5 long description.

The sequences were manually inspected and edited using BioEdit version 7.2.5 (Hall, 1999). Phylogenetic analysis was conducted using maximum likelihood in MEGA-12 (Kumar et al., 2024), with sequence alignment performed by ClustalX 2.1 (Thompson et al., 2002). Best-fit nucleotide substitution models were determined for each marker: the Tamura et al. (2013) model with an additional parameter accounting for a proportion of invariable sites (+I) for 18S rRNA and Tamura-Nei (TN93) model with a discrete gamma distribution (+G) for *COI*. Phylogenetic trees were constructed using 1000 bootstrap replicates to ensure analytical robustness (Hall, 1999; Thompson et al., 2002; Tamura et al., 2013). The *COI* and 18S rRNA analyses incorporated sequences from all available species within *Kalicephalus. Angiostrongylus cantonensis* was used as the outgroup for each marker. Genetic distances were calculated in MEGA-12, using pairwise distance (*p*-distance) to assess genetic differences between species, between genera and within species. All sequences were retrieved from GenBank (see Figure 5).

## Results

### Taxonomy

Phylum: Nematoda Diesing, 1861

Class: Chromadorea Inglis, 1983

Order: Rhabditida Chitwood, 1933

Family: Diaphanocephalidae Travassoss, 1920

Genus: *Kalicephalus* Molin, 1861

Subgenus: *Occipitodontus* (Ortlepp, 1923)

Species: *Kalicephalus colossus* sp. nov. Charoennitiwat, 2026 (Table 1, Figures 1 and 2)

**Type-host:** *Naja kaouthia* Lesson, 1831.

**Type-locality:** Bangkok’s suburbs (e.g., Lak Si [13°53′14.3″ N, 100°35′31.7″ E], Don Mueang [13°54′50.1″ N, 100°35′23.8″ E] and Prawet [13°43′20.7″ N, 100°40′53.8″ E]) in Thailand served as collection sites; however, the exact coordinates of individual hosts were not recorded.

**Collection date:** 23 February 2021 to 20 June 2025.

**Site of infection:** Oesophagus

**Parasite intensity:** 1–48 (on average 13) nematodes in the 78 hosts examined

**Prevalence:** 18% (14 infected hosts)

**Abundance:** 2 parasites per host (total parasites recovered: 174 individuals)

**ZooBank LSID:** urn:lsid:zoobank.org:pub:0DE846E4-FE3A-4540-9D52-29546B6F82B4

**Etymology:** The specific epithet *colossus* is derived from the Latin word, meaning a gigantic or something of enormous size. The name refers to the comparatively large body size of this nematode species relative to other described species from the genus.

#### General description

Body cylindrical, both extremities gradually tapering, width at base of buccal capsule lesser than maximum width of body (Figure 1A,B). Cuticle finely transversely striated (Figure 3A). Cephalic inflation present (Figures 1C, 2A,B). Anterior end curved, not tilted; with rudimentary corona radiata and cuticular corners of head very inflated to angular (Figures 1C, 2A, 3B). Buccal capsule well developed, bivalved, composed of 2 lateral valves formed by several chitinoid elements (Figures 1C, 2A,B, 3C). Oral opening narrow, dorsoventrally elongated. Lip surfaces bearing frilled corona radiata (Figure 3C). Anterior chitinoid ridge curved and narrow (Figures 1C, 2B). Posterior chininoid pieces with prolongation extending posterior to anterior end of oesophagus (Figures 1C, 2A,B). Dorsal gutter relatively short with wide tip. Oesophagus expanded anteriorly, variable otherwise, short and heavily bulbed to elongate and moderately bulbed (Figures 1C, 2A,B). Nerve ring located at mid-region of oesophagus and excretory pore situated between the level of the nerve ring and the widest level of oesophageal bulb (Figures 1A–C, 2B, 3D). Long, fine cervical papillae located shortly posterior nerve ring (Figures 3C, 3E). Intestine straight, with rectal glands at its posterior extremity (Figures 1G, 2D).

Males (based on all 15 specimens): body length 13 345–17 680, maximum width 582–703. Buccal capsule 204–271 deep, head diameter 376–456. Oesophagus 634–850 long, maximum width of the bulb region 250–312 and at oesophago–intestinal junction 139–192. Oesophagus end, excretory pore, nerve ring and cervical papillae situated at 891–1075, 468–601, 387–493, 526–705 from anterior end. Testis single, tubular, flexing posteriorly in anterior region. Bursa with long narrow ventral ray that diverges distally (Figures 1D,E, 2E,F, 3F). Lateral rays with externolateral short and thumb-like, while medio- and postero-lateral are longer and finely pointed (Figures 1D,E, 2E,F). Externodorsals thumb-like (Figures 1D,E, 2E,F). Terminal branches of dorsal ray of pattern III (according to Schad, 1962) (Figure 1E). Dorsal bursal ray with all branches closer together than to externodorsals (Figures 1D,E, 2E,F). Body cuticle width 22–39. Spicules 545–716 long, maximum width 18–35 and equal in size (Figures 1D, 2E,F), covered with sheath with fine transversal striations. Gubernaculum 101–146 long, navicular in shape (Figures 1D, 2F). Telamon 56–95 long, maximum width 17–54 with crests in lateral view, genital cone well-developed.

Gravid females (based on all 15 specimens): body length 14 854–21 159, maximum width 680–903. Buccal capsule 227–285 deep, head diameter 451–503. Oesophagus 738–920 long, maximum width of the bulb region 265–342 and at oesophago–intestinal junction 132–221. Oesophagus end, excretory pore, nerve ring and cervical papillae located at 990–1165, 537–720, 447–604 and 601–771 from the anterior end. Amphidelphic, vulva (Figures 1F, 2C, 3G) closer to mid-body than to anus (Figures 1B, 3H), with a vulvar ratio (prevulvar: postvulvar body length) of 1.7–2.1. Vagina short, bifurcating into ovejectors and short, muscular sphincters (Figures 1F, 2C). Body cuticle width 23–45. Eggs at morula stage in uterus and vagina, with a smooth, thin and elliptical shell 57–88 long and 41–54 wide. Rectal glands at final portion of intestine. Anus with posterior lip more prominent (Figures 1G, 2D, 3H). Tail elongated, 311–498 long, tapering posteriorly, with a terminal spine (Figures 1G, 2D, 3H).

### Type materials

Holotype: Adult male, MUMNH-NEM0036 (specimen code SN152M4), collected from the oesophagus of *N. kaouthia* (project ID SN152; AASLab ID AAS169 [EL-Nk-055]) on 20 June 2025, Thailand; coll. Vachirapong Charoennitiwat and team, at Applied Animal Science Laboratory (AASLab), Department of Biology, Faculty of Science, Mahidol University. Allotype: Gravid female, MUMNH-NEM0037 (SN11F1), collected from *N. kaouthia* (SN011; AAS011 [EL-Nk-009]) on 23 February 2021, Thailand; same collectors. Paratypes: 1 male, MUMNH-NEM0038 (SN11M1), collected from the same host and on the same date as the allotype; 1 female, MUMNH-NEM0039 (SN152F4), collected from the same host and on the same date as the holotype; same locality and collectors. All type specimens are deposited in the Mahidol University Museum of Natural History (MUMNH), Faculty of Science, Mahidol University, Thailand. Remarks: Measurements of all examined specimens, including type and non-type materials, are provided in Supplementary Table S1. Their morphological characteristics are consistent with the general description.

### Remarks

Based on Anderson et al. (2009), *K. colossus* sp. nov. is assigned to the genus *Kalicephalus* Molin, 1861, and morphologically corresponds to the subgenus *Occipitodontus* Ortlepp, 1923, based on the following combination of characters: peri-oral groove delimited only by an anterior ridge; buccal cavity lacking membranous processes at its base (Figure 3C); buccal capsule with prominent posteriorly directed dorsal and ventral projections; rudimentary corona radiata present (Figures 1C, 2A,B); spicules alate, equal in length (Figures 1D, 2E,F); copulatory bursa lobate, without a muscular prebursal bulge; dorsal bursal ray with all branches closer together than to the externodorsal rays (Figures 1D,E, 2E,F); females usually amphidelphic; vulva located closer to the mid-body than to the anus in mature specimens (Figures 1B, 1F, 2C); parasites inhabiting the digestive tract other than the rectum. These characters are consistent with the diagnosis of *Occipitodontus* and distinguish the species from other subgenera of *Kalicephalus*. In addition, *K. colossus* sp. nov. is currently known only from Thailand and infects *N. kaouthia*.

Among species traditionally assigned to *Occipitodontus* (Anderson et al., 2009), *K. colossus* sp. nov. differs from *K. fimbriatus* in lacking the strongly developed posteriorly prolonged dentigerous plates extending deeply into the anterior oesophagus. In addition, *K. colossus* sp. nov. possesses a substantially larger body size in both males (13 345–17 680 vs 9520–12 000) and females (14 854–21 159 vs 11 400–14 630), greater body width in both sexes (♂ 582–703 vs 400–440; ♀ 680–903 vs 460–570), deeper buccal capsule (♂ 204–271 vs 230–260; ♀ 227–285 vs 230–280) and longer spicules (545–705 vs 520) than *K. fimbriatus* (Table 1). The 2 species also differ in site of infection, with *K. fimbriatus* reported from the stomach, whereas *K. colossus* sp. nov. inhabits the proximal oesophagus. *Kalicephalus fimbriatus* is documented from India and Malaya and primarily infects the banded krait (*Bungarus fasciatus*) and the Sunda king cobra (*Ophiophagus bungarus*), whereas *K. colossus* sp. nov. infects *N. kaouthia* in Thailand.

*Kalicephalus colossus* sp. nov. resembles *K. assimilis* in lacking strongly prolonged dentigerous plates but differs in its markedly larger body size (♂ 13 345–17 680 vs 6290–8790; ♀ 14 854–21 159 vs 7350–10 690), greater maximum body width (♂ 582–703 vs 230–450; ♀ 680–903 vs 320–450), deeper buccal capsule (♂ 204–271 vs 110–150; ♀ 227–285 vs 140–190), larger dorsoventral head diameter (♂ 376–456 vs 160–290; ♀ 451–503 vs 300–350), longer oesophagus (♂ 634–850 vs 470–580; ♀ 738–920 vs 500–590) and longer spicules (545–705 vs 410–550) (Table 1). The vulvar ratio of *K. colossus* sp. nov. (pre-vulvar to post-vulvar ratio 1.67–2.12) overlaps only partially with that reported for *K. assimilis. Kalicephalus assimilis* has been recorded from Indonesia, Malaya and China infecting *O. Bungarus*, whereas *K. colossus* sp. nov. infects *N. kaouthia* in Thailand.

Among currently recognized congeners, *K. colossus* sp. nov. is morphologically most similar to *K. giganteus*, particularly in overall body size and oesophageal dimensions. However, the 2 species can be readily distinguished by the morphology of the posterior chitinoid plates of the buccal capsule. In *K. colossus* sp. nov., these projections are elongate and narrow, whereas in *K. giganteus* they are short, rounded and stump-like. The species further differ in the position of the excretory pore, which is situated near the level of the nerve ring in *K. colossus* sp. nov. (♂ 468–604; ♀ 537–720) but distinctly posterior, below the oesophago–intestinal junction, in *K. giganteus* (♂ 680–960; ♀ 790–980). Corresponding differences are also evident in the relative positions of the cervical papillae from the anterior extremity (♂ 526–705 vs 710–1060; ♀ 601–771 vs 830–1070, respectively) (Table 1). In addition, *K. giganteus* is currently known from New Guinea infecting the Papuan python (*Liasis papuanus*). The combination of its distinctive buccal morphology, exceptionally large body size, oesophageal localization and distinct host association clearly supports the recognition of *K. colossus* sp. nov. as a distinct species within the subgenus *Occipitodontus*.

### Variation

Based on the 5 hosts examined, the number of *K. colossus* sp. nov. individuals per host ranged from 4 to 48 worms (mean: 18) and showed considerable morphological variation, with females generally larger than males (Supplementary Table S1). To assess morphological homo- and heterogeneity in the absence of defined populations or strains, all individuals were analysed using 12 shared morphological characters (Supplementary Table S1). PCA revealed no discrete morphological clustering among individuals. In the 2D PCA of PC1 and PC2, all specimens formed a single, broadly overlapping group, with extensive overlap of convex hulls and no clear group boundaries (Figure 4A). The first 2 principal components together explained 72.09% of the total variance, with a marked decline in eigenvalues from PC1 (57.52%) to PC2 (14.57%), indicating that most morphological variation is structured along a single major axis rather than reflecting distinct morphotypes. When sex was included as a grouping factor, the same PCA revealed clear differentiation between males and females (Figure 4B). Male and female individuals formed 2 partially non-overlapping groups, each enclosed by distinct convex hulls, demonstrating pronounced sexual dimorphism. DA further showed that body length was the main variable contributing to overall individual variation, whereas cuticle width was the primary measurement responsible for separating males from females.

### Genetic characterization and phylogenetic position

Molecular characterization of *K. colossus* sp. nov., based on partial sequences of the nuclear 18S rRNA and mitochondrial *COI* genes, included phylogenetic analyses using available *Kalicephalus* sequences from GenBank together with representative strongylid nematodes (Figure 5A,B). In the 18S rRNA phylogeny (Figure 5A), *K. colossus* sp. nov. formed a strongly supported clade (bootstrap = 99) and clustered most closely with *K. bungari*. This group was recovered together with a GenBank sequence deposited as ‘*Kalicephalus cristatus*’. The sampled *Kalicephalus* taxa formed a distinct cluster separated from the remaining strongylid nematodes included in the analysis. Branch support within the *Kalicephalus* clade was generally high for the major nodes recovered in the 18S topology.

In the *COI* phylogeny (Figure 5B), *K. colossus* sp. nov. formed a strongly supported monophyletic lineage (bootstrap = 100) and was recovered closest to *K. bungari*. However, the sampled *Kalicephalus* taxa were not recovered as monophyletic because *K. indicus* clustered separately with other strongylid taxa rather than with the remaining *Kalicephalus* species. Relationships among several internal nodes in the *COI* tree received only low to moderate bootstrap support. Nevertheless, the sequences of *K. colossus* sp. nov. consistently formed a distinct lineage in both phylogenetic reconstructions.

Pairwise genetic divergence between *K. colossus* sp. nov. and other available *Kalicephalus* species ranged from 1.1 to 1.4% for 18S rRNA and 12 to 13% for *COI*. The lowest 18S divergence was observed between *K. colossus* sp. nov. and the sequence deposited as ‘*K. cristatus*’ (1.1%), whereas the lowest *COI* divergence was observed with *K. bungari* (12%). In contrast, greater genetic divergence was observed between *K. colossus* sp. nov. and *K. indicus* in both markers.

### Natural history

*Kalicephalus colossus* sp. nov. was detected in 14 of 78 examined monocled cobras (18% prevalence), with infection intensities ranging from 1 to 48 worms per host (mean intensity ≈ 13). This species was found exclusively in the oesophagus and was frequently observed in co-infections with *Paracapillaria najae* (*sensu* Charoennitiwat et al., 2023), *K. bungari* and an undescribed *Kalicephalus* species (ongoing investigations).

The hookworms typically formed dense aggregations within the oesophageal mucus and were mainly distributed in the proximal region of the oesophagus. In heavily infected individuals, the parasites were sometimes visible upon opening the mouth of the hosts. The presence of blood within the digestive tract of the worms suggests that *K. colossus* sp. nov. feeds on host blood (Supplementary Fig. S1). Despite relatively high parasite burdens in some hosts, no obvious gross lesions or apparent tissue damage were observed during necropsy examinations. Although precise geographic coordinates for the collection sites were not available, all infected cobras originated from suburban areas surrounding Bangkok.

## Discussion

The discovery of *K. colossus* sp. nov. in the monocled cobra, *N. kaouthia*, from central Thailand contributes additional information on the diversity, host associations and possible ecological differentiation of reptile hookworms in the Oriental region. Beyond its taxonomic novelty, this species provides additional context for understanding how morphological variation and host ecology may influence parasite diversification within *Kalicephalus*.

### Taxonomy and phylogenetic relationships in *Kalicephalus*

Although *Kalicephalus* is morphologically well defined at the generic and subgeneric levels, species delimitation has historically relied on a limited set of characters, particularly buccal capsule morphology, spicule length and body size (e.g., Schad, 1962; Lichtenfels, 1980; Baker, 1987; Anderson et al., 2009). These characters remain diagnostically informative and support the placement of *K. colossus* sp. nov. within *Occipitodontus* based on its distinctive posteriorly directed chitinoid plates (*sensu* Anderson et al., 2009). However, recent studies have questioned whether traditional subgeneric classification accurately reflects evolutionary relationships within *Kalicephalus* (Ahmed et al., 2022; Haick et al., 2025). Consequently, a comprehensive revision of the genus integrating morphology, morphometrics, molecular phylogenetics, host associations and ecological data is likely required to better resolve species boundaries and higher-level systematic relationships (Keating et al., 2023).

Although 34 species of *Kalicephalus* have been described, most remain characterized primarily on the basis of morphology, and molecular sequence data are currently available for only a limited number of taxa. In addition, available molecular data are often restricted to single-marker sequences, with inconsistent gene representation among species, thereby limiting overlap among datasets and constraining comprehensive phylogenetic assessment within the genus. Consequently, the evolutionary validity of the currently recognized subgenera remains difficult to evaluate (e.g., Haick et al., 2025). Expanding taxon sampling and incorporating additional nuclear and mitochondrial markers will therefore be important for resolving phylogenetic relationships and testing the systematic framework currently based largely on morphology.

The nuclear 18S rRNA dataset recovered *K. colossus* sp. nov. as a distinct lineage closely associated with *K. bungari*, with both taxa clustering together with a GenBank sequence deposited as ‘*K. cristatus*’, although the taxonomic assignment of this sequence remains uncertain because the corresponding species designation has not been clearly established in the published literature (*sensu* Chilton et al., 2006). The relatively conserved nature of the nuclear gene resulted in low genetic divergence among sampled taxa, but the marker nevertheless clearly separated *K. colossus* sp. nov. from other available *Kalicephalus* sequences (Chan et al., 2020; Thaenkham et al., 2022). The 18S topology also recovered the sampled *Kalicephalus* taxa as a monophyletic group relative to the other strongylid nematodes included in the analysis. However, interpretation of subgeneric relationships remains limited because the sampled taxa currently represent different putative subgenera, including *Occipitodontus, Kalicephaloides* (*K. bungari*) and possibly *Schadius*. The latter inference is tentative because the GenBank sequence deposited as ‘*K. cristatus*’ (Chilton et al., 2006) may represent *K. costatus*, a species historically assigned to *Schadius*. Consequently, the current topology does not provide clear support for subgeneric differentiation within *Kalicephalus*.

In contrast, the mitochondrial *COI* dataset provided less stable phylogenetic resolution at the generic level. Although *K. colossus* sp. nov. formed a distinct and strongly supported lineage together with *K. bungari*, the sampled *Kalicephalus* taxa were not recovered as monophyletic because *K. indicus* clustered separately among other strongylid taxa. A similar pattern was reported by Zhou et al. (2019), in which *K. indicus* showed close affinity to *Ancylostoma ceylanicum*. Several factors may contribute to this pattern, including limited taxon sampling, the use of a single mitochondrial marker, rapid evolutionary rates of *COI* sequences, substitution saturation and possible inconsistencies in sequence identification among publicly available datasets (Blouin, 2002; Nadler and de León, 2011). Mitochondrial genes are often highly informative for species-level identification in nematodes because of their elevated mutation rates, but single-locus mitochondrial phylogenies may provide unstable or misleading resolution for deeper evolutionary relationships and higher taxonomic levels when taxon sampling remains limited (Blouin, 2002; Hu et al., 2004).

### Morphological and functional considerations

From a comparative morphological perspective, one of the most distinctive features of *K. colossus* sp. nov. is its exceptional body size relative to other members of *Occipitodontus* and related congeners. Body size in parasitic nematodes is often associated with microhabitat use and feeding strategy, reflecting trade-offs between attachment efficiency, fecundity and host exploitation (Arneberg et al., 1998; Morand and Poulin, 2002). The markedly deep buccal capsule and elongated posterior chitinoid elements in *K. colossus* sp. nov. may be compatible with attachment within the oesophageal environment, which is subjected to swallowing movements and muscular contractions, although similar structures are also present in other *Kalicephalus* species occupying different regions of the digestive tract.

The localization of *K. colossus* sp. nov. in the anterior part of the oesophagus contrasts with the gastric or intestinal occurrence reported for other morphologically similar species traditionally assigned to *Occipitodontus* species: *K. fimbriatus* (stomach) and *K. assimilis* (intestine) (Schad, 1962; Purwaningsih, 2011), indicating variation in microhabitat occupancy within the digestive tract among related species. However, given the limited number of species and the absence of experimental or biomechanical data, it remains unclear whether these morphological differences reflect specific adaptive specializations or simply represent lineage-specific variation. The combination of large body size and robust buccal structures may relate to attachment and feeding within the oesophageal environment, whereas the elongated spicules likely reflect reproductive morphology rather than feeding adaptations (e.g., Aznar et al., 2018).

### Biogeography and host associations

In terms of geographic distribution, *Kalicephalus* species exhibit a predominantly tropical and subtropical range, with the highest diversity reported from the Oriental and Neotropical regions (Schad, 1962; Zhang et al., 2011; Kuzmin et al., 2013; Chandel and Dolma, 2025; Haick et al., 2025). The discovery of *K. colossus* sp. nov. in central Thailand, together with species currently under study (*sensu* Ratnarathorn et al., 2025), supports the hypothesis that Southeast Asia represents a major centre of diversification for reptile hookworms. However, current distribution patterns are likely strongly biased by uneven sampling effort, as many regions in Southeast Asia and reptile host taxa worldwide remain poorly surveyed. Consequently, the apparent endemism of several species, including *K. colossus* sp. nov., should be interpreted cautiously until broader geographic and host-based sampling is conducted.

If future studies confirm a restricted host association in *K. colossus* sp. nov., its distribution may reflect aspects of host ecology and regional dispersal history. Parasites exhibiting relatively narrow host associations may show spatial distributions that reflect host population structure and dispersal history (Hoberg and Brooks, 2008; Blasco-Costa and Poulin, 2013). In this context, *Kalicephalus* species may serve as informative biological markers for reconstructing host biogeography and ecological connectivity across Southeast Asia. Future studies integrating parasite phylogenies with host population genetics may therefore provide novel insights into regional host–parasite associations and biogeographic patterns (e.g., Blasco-Costa and Poulin, 2013).

### Host ecology, life cycle strategy and urban transmission

The monocled cobra (*N. kaouthia*) is a large, mobile and ecologically flexible predator that occupies a wide range of habitats, from natural wetlands to heavily urbanized landscapes (Cox et al., 2012; Ratnarathorn et al., 2019, 2024, 2026). Such ecological flexibility may increase exposure to diverse transmission environments, prey types and parasite communities. The exclusive occurrence of *K. colossus* sp. nov. in *N. kaouthia* may indicate a relatively narrow host association, although additional sampling across sympatric snake species is required to evaluate host specificity and possible host-associated ecological differentiation (Šimková, 2024).

More broadly, host diversity and ecological opportunity have been proposed as important drivers of parasite diversification (Kamiya et al., 2014; Ratnarathorn et al., 2025). Compared with other morphologically similar *Kalicephalus* species traditionally assigned to *Occipitodontus* and reported from elapid snakes, *K. colossus* sp. nov. infects a host exhibiting broadly comparable ecological characteristics, including terrestrial habits and frequent contact with soil-associated environments. This observation suggests that ecological factors such as habitat use and transmission opportunity may contribute to host association patterns within *Kalicephalus*. Parasite diversification within the genus may therefore be influenced not only by host lineage but also by ecological opportunity, although broader comparative sampling is needed to evaluate these relationships (e.g., Poulin and Morand, 2000; Hoberg and Brooks, 2008; Blasco-Costa and Poulin, 2013).

Although precise geographic coordinates were not available for individual collection sites, all infected cobras originated from suburban areas surrounding Bangkok. The life cycle of *K. colossus* sp. nov. remains unknown; however, as a member of Strongylida, the species likely possesses a direct life cycle involving environmental development of infective larval stages, as reported for many strongylid nematodes (Morand et al., 2006; Gordon et al., 2017). This pattern is consistent with the frequent detection of infected cobras in humid, human-modified environments where snakes commonly occur, including gardens, drainage systems, rice fields and construction sites (Ratnarathorn et al., 2024, 2026), which may facilitate the persistence of free-living larval stages (e.g., Charoennitiwat et al., 2025a).

From an ecological viewpoint, *K. colossus* sp. nov. may occur successfully in human-modified environments where suitable hosts, elevated host density and favourable environmental conditions are present. This aligns with growing evidence that urbanization can facilitate parasite persistence and transmission in wildlife, particularly for parasites with direct life cycles (Bradley and Altizer, 2007; Werner and Nunn, 2020; Ratnarathorn et al., 2025). In this sense, *K. colossus* sp. nov. may represent part of the helminth fauna persisting in suburban environments associated with human-modified ecosystems (Mackenstedt et al., 2015). This interpretation is tentatively supported by the lack of detection of *K. colossus* sp. nov. in snakes examined from relatively less urbanized areas (e.g., Saraburi–Ayutthaya) and its comparatively higher prevalence in samples from the Bangkok metropolitan area.

### Host condition and potential pathogenic effects

Despite relatively high intensities in some individuals, infections with *K. colossus* sp. nov. were not associated with obvious gross lesions during necropsy examinations. This observation may indicate limited overt pathogenic effects under natural conditions, although histopathological and physiological studies are required to evaluate host responses (Råberg et al., 2007). Such tolerance is often interpreted as a hallmark of long-term host–parasite coevolution, in which excessive parasite virulence would be counter-selected due to reduced host survival and transmission opportunities (Råberg, 2014).

Previous reports have documented severe clinical signs associated with *Kalicephalus* infections in captive snakes (e.g., Chaiyabutr and Chanhome, 2002; Vasaruchapong et al., 2017), where stress, crowding and immunosuppression may disrupt host–parasite equilibrium. This highlights the importance of distinguishing observations from wild populations from pathological outcomes observed under captive conditions. In wild populations, *K. colossus* sp. nov. may appear to represent a persistent component of the helminth community in *N. kaouthia* without obvious gross pathological effects.

### Broader implications for parasite biodiversity

The discovery of *K. colossus* sp. nov. reinforces the view that parasite diversity in Southeast Asia remains substantially underestimated, even in relatively well-studied hosts such as medically important snakes (e.g., Charoennitiwat et al., 2023, 2024a, 2025a, 2025b). Given the long and complex taxonomic history of *Kalicephalus*, including repeated species reallocations, synonymies and revisions (e.g., Schad, 1962), together with the limited availability of molecular data, additional cryptic diversity within the genus is likely to remain undocumented, particularly in tropical regions with high reptile richness (Raven et al., 2020). Documenting parasite diversity remains important for understanding host–parasite associations and the broader biodiversity of tropical ecosystems, contributing to energy flow, population regulation and host immune evolution (Poulin and Morand, 2000; Pedersen and Fenton, 2007; Hatcher et al., 2012). Such studies also provide essential baseline data for future systematic, ecological and evolutionary investigations.

## Conclusion

The present study formally describes *Kalicephalus colossus* sp. nov., a new hookworm species parasitizing the oesophagus of *Naja kaouthia* in central Thailand. Integrative evidence from morphology, SEM observations, morphometric analyses and molecular phylogenetics supports its recognition as a distinct species within the group traditionally assigned to the subgenus *Occipitodontus*. The species is distinguished by its exceptionally large body size, characteristic posterior chitinoid projections of the buccal capsule and distinctive combination of reproductive and cephalic characters. Phylogenetic analyses further highlight the need for broader systematic revision within *Kalicephalus*, as current morphology-based subgeneric classifications remain insufficiently tested using molecular data. The discovery of *K. colossus* sp. nov. also reinforces the view that Southeast Asia represents an important centre of reptile hookworm diversity. More broadly, this study demonstrates the continuing value of integrative taxonomy for documenting parasite biodiversity, clarifying host–parasite associations and improving understanding of nematode evolution in tropical ecosystems.

## Supporting information

10.1017/S0031182026102248.sm001 Charoennitiwat et al. supplementary material 1 Charoennitiwat et al. supplementary material

10.1017/S0031182026102248.sm002 Charoennitiwat et al. supplementary material 2 Charoennitiwat et al. supplementary material

10.1017/S0031182026102248.sm003 Charoennitiwat et al. supplementary material 3 Charoennitiwat et al. supplementary material

## Data Availability

The data supporting the findings of this study are provided as supplementary files accompanying this article on the journal’s publication platform.

## Supplementary material

The supplementary material for this article can be found at https://doi.org/10.1017/S0031182026102248.

## Figure 4 Long description

The image A shows a scatter plot representing principal component analysis of specimens. The horizontal axis is labeled PC1 57.523 percent and the vertical axis is labeled PC2 14.579 percent. The plot contains black dots representing individual specimens, clustered within a single polygon, indicating a general distribution without distinct separation. The image B shows a scatter plot with the same axis labels as image A. This plot illustrates clustering by sex, with two distinct polygons. Pink dots and lines represent female specimens, while blue dots and lines represent male specimens. The separation of these clusters suggests differentiation based on sex. The plot highlights how specimens group differently along the principal components, with no significant outliers noted.

Navigate back to Figure 4.

## Figure 5 Long description

The image shows two phylogenetic trees labeled A and B. Tree A is based on the 18S rRNA gene, displaying relationships among various strongylid species. The tree is oriented vertically with branch lengths indicating the number of substitutions per site. Bootstrap values are shown at nodes, representing support from maximum likelihood and Bayesian inference. Kalicephalus colossus is highlighted in red, with sequences PX939515 and PX939516. Other species include Kalicephalus bungari, Kalicephalus cristatus, Necator americanus, Oesophagostomum aculeatum, Strongylus equinus and Angiostrongylus cantonensis. Tree B is based on the COI gene, also oriented vertically. Kalicephalus colossus is again highlighted in red with sequences PX940603 and PX940605. Other species include Kalicephalus bungari, Ancylostoma duodenale, Kalicephalus indicus, Necator americanus, Oesophagostomum columbianum and Angiostrongylus cantonensis. Bootstrap values are present at nodes, indicating support levels. Both trees use Angiostrongylus cantonensis as an outgroup.

Navigate back to Figure 5.

## Table 1 Long description

The table lists host, locality, infection organ, sample sizes, and measurement ranges for male and female worms across five Kalicephalus species entries from different references. Species compared are Kalicephalus colossus, Kalicephalus fimbriatus, Kalicephalus assimilis reported in two sources, and Kalicephalus giganteus. Hosts and sites differ: colossus is from Naja kaouthia in the oesophagus in Thailand, fimbriatus is from Bungarus fasciatus and Ophiophagus bungarus in the stomach in India and Malaya, and the assimilis and giganteus records are intestinal from Ophiophagus bungarus or Liasis papuanus across multiple regions. Body length ranges show females are consistently longer than males within each species, with giganteus and colossus generally the largest and assimilis generally the smallest. Male spicule length is notably greatest in giganteus, while colossus and fimbriatus are intermediate and assimilis is shorter. Several measurements are missing for some literature sources, and one assimilis column has no male or female sample size, so comparisons should be treated as approximate and dependent on the original study methods.

Navigate back to Table 1.
