# Supplementary material for: Kalicephalus (Occipitodontus) colossus (Nematoda: Diaphanocephalidae): a new hookworm from Naja kaouthia in central Thailand
Source: Parasitology. 2026 Jun 16;153(7):938–51. doi: 10.1017/S0031182026102248 (PMC13735555; doi:10.1017/S0031182026102248)
Supplement: Supplementary file 1 [file S0031182026102248sup001.docx]

**Figure S1**: Gross appearance of *Kalicephalus colossus* sp. nov. observed in the oesophagus of freshly dissected cobras.
