# Supplementary material for: Kalicephalus (Occipitodontus) colossus (Nematoda: Diaphanocephalidae): a new hookworm from Naja kaouthia in central Thailand
Source: Parasitology. 2026 Jun 16;153(7):938–51. doi: 10.1017/S0031182026102248 (PMC13735555; doi:10.1017/S0031182026102248)
Supplement: Supplementary file 2 [file S0031182026102248sup003.docx]

**Table S2:** Specific primers, thermal cycling conditions, and PCR amplification details for the molecular investigation of *Kalicephalus* *colossus* sp. nov.

| **Marker** | **Primer** | **Primer sequence** | **Length (bp)** | **Denaturation** | **Extension** | **Reference** |
| --- | --- | --- | --- | --- | --- | --- |
| 18S rRNA | 1096F | 5′-GGT AAT TCT GGA GCT AAT AC–3′ | 800  (partial) | 94 °C 5 min, 5 cycles of 94 °C 30 sec, 45 °C 30 sec 72 °C 70 sec, 35 cycles of 94 °C 30 sec, 54 °C 30 sec 72 °C 70 sec | 72 °C 5 min | Holterman *et al*., 2006 |
|  | 1916R | 5′-TTT ACG GTC AGA ACT AGG G-3′ |  |  |  |  |
| *COI* | COI_C345_F | 5′-GGA RCA TAT RAG TTT ATT TGT-3′ | 771 | 95 °C 5 min, 35 cycles of 95 °C 30 sec, 52 °C 1 min, 72 °C 45 sec | 72 °C 10 min | In house primers |
|  | COI_C345_R | 5′-CCA TGT AAW CCW GCA AAA TG-3′ | (partial) |  |  |  |

**Holterman M, van der Wurff A, van den Elsen S, van Megen H, Bongers T, Holovachov O, Bakker J and Helder J** (2006) Phylum-wide analysis of SSU rDNA reveals deep phylogenetic relationships among nematodes and accelerated evolution toward crown clades. *Molecular Biology and Evolution* **23**, 1792–1800. doi: 10.1093/molbev/msl044.
